# A better scoring model for de novo peptide sequencing: the symmetric difference between explained and measured masses

**DOI:** 10.1186/s13015-017-0104-1

**Published:** 2017-05-11

**Authors:** Thomas Tschager, Simon Rösch, Ludovic Gillet, Peter Widmayer

**Affiliations:** 1 Department of Computer Science, ETH Zurich, Universitätstrasse 6, 8092 Zurich, Switzerland; 2 Department of Biology, ETH Zurich, Auguste-Piccard-Hof 1, 8093 Zurich, Switzerland

**Keywords:** Computational proteomics, Peptide identification, De novo peptide sequencing, Mass spectrometry

## Abstract

**Background:**

Given a peptide as a string of amino acids, the masses of all its prefixes and suffixes can be found by a trivial linear scan through the amino acid masses. The inverse problem is the *ideal* *de novo* *peptide sequencing problem*: Given all prefix and suffix masses, determine the string of amino acids. In biological reality, the given masses are measured in a lab experiment, and measurements by necessity are noisy. The (real, noisy) *de novo peptide sequencing problem* therefore has a noisy input: a few of the prefix and suffix masses of the peptide are missing and a few other masses are given in addition. For this setting, we ask for an amino acid string that explains the given masses as accurately as possible.

**Results:**

Past approaches interpreted accuracy by searching for a string that explains as many masses as possible. We feel, however, that it is not only bad to not explain a mass that appears, but also to explain a mass that does not appear. We propose to minimize the symmetric difference between the set of given masses and the set of masses that the string explains. For this new optimization problem, we propose an efficient algorithm that computes both the best and the *k* best solutions. Proof-of-concept experiments on measurements of synthesized peptides show that our approach leads to better results compared to finding a string that explains as many given masses as possible.

**Conclusions:**

We conclude that considering the symmetric difference as optimization goal can improve the identification rates for de novo peptide sequencing. A preliminary version of this work has been presented at WABI 2016.

**Electronic supplementary material:**

The online version of this article (doi:10.1186/s13015-017-0104-1) contains supplementary material, which is available to authorized users.

## Background

The determination of the amino acid sequence of a peptide based on mass spectrometric data is an important task in proteomics. A typical tandem mass spectrometry experiment consists of three steps [1, 2]. First, the mass spectrometer measures the mass-to-charge ratio and the abundance of the analyzed peptide. Second, the peptide of interest is selected by the instrument to perform fragmentation, i.e. dissociation of multiple copies of this peptide at random positions into charged prefix and suffix fragments. Finally, the mass spectrometer measures the mass-to-charge ratios and abundances of the resulting fragments. Afterwards, data processing algorithms deconvolute mass-to-charge ratios to masses. There are several sources of uncertainties and errors in every step of this experiment. Therefore, some masses of prefix and suffix fragments are missing, while other masses are given in addition.

In this noisy setting, *de novo sequencing* is the problem to compute as accurately as possible the amino acid string of the recorded peptide given the mass *M* of the peptide measured in the first step of the experiment and the set *X* of prefix and suffix masses measured in the third step. Several approaches [2–5] tackle this problem by computing an amino acid string $\texttt {S}$ with mass *M*, such that the set $\text{TS}(\texttt {S})$ of all prefix and suffix masses of $\texttt {S}$ contains as many masses as possible of the set *X*. This scoring model is often referred to as *shared peaks count*. Besides only considering the size of the intersection $\text{TS}(\texttt {S}) \cap X$, several of these approaches [6–8] can also maximize a more elaborate score on the masses in $\text{TS}(\texttt {S}) \cap X$.

However, considering only the intersection of $\text{TS}(\texttt {S})$ and *X* might lead to a bias towards the use of amino acids with small masses. For example, the amino acid glutamine has the same mass as the sum of the masses of a glycine and an alanine. When maximizing $|\text{TS}(\texttt {S}) \cap X|$, one can always replace a glutamine by both a glycine and an alanine in the string $\texttt {S}$ without decreasing the size of the intersection. In an ideal experiment, where all prefix and suffix masses and no other masses are given in *X*, there exists a string $\texttt {S}$ with $\text{TS}(\texttt {S}) = X$. However, in a real-world experiment with missing masses, we want to explain masses that are in *X*, but not to explain masses that are not in *X*. Dančík et al. [6] noted this problem and proposed a probabilistic scoring model incorporating penalty scores for some specific fragment masses present in $\text{TS}(\texttt {S})$ but not in *X*. However, current algorithms do not systematically account for exactly those masses in $\text{TS}(\texttt {S})\setminus X$.

In this paper, we propose a new fundamental scoring model that considers both the masses in $\text{TS}(\texttt {S}) \cap X$ and the masses in $\text{TS}(\texttt {S}) \setminus X$. Conceptually, our aim is to minimize the size of the symmetric difference $|\text{TS}(\texttt {S})\, \Delta\, X| = |\text{TS}(\texttt {S}) \setminus X| + |X \setminus \text{TS}(\texttt {S}) |$ instead of maximizing the size of the intersection $|\text{TS}(\texttt {S}) \cap X|$, namely the shared peaks count. We explore this scoring model by first giving a precise definition of our new optimization problem and by developing an algorithm for this problem. Then, we provide a proof-of-concept implementation and study how the symmetric difference improves over the shared peaks count in terms of quality of the result. Our experiments demonstrate that the symmetric difference scoring model leads to higher identification rates that do not come at an unbearable computational cost. We hope that our results encourage software developers to integrate the proposed scoring model in commercial or advanced open-source de novo sequencing software in the future.

The paper is structured as follows. In section “Problem definition” we precisely define the considered de novo sequencing problem. In section “Algorithm”, we develop a dynamic programming algorithm to find the best and the *k* best strings with respect to our objective function. We first describe a simplified variant that does not consider different types of fragments and molecular losses that can happen during the fragmentation process. Then, we describe how we can additionally compute the *k* best strings and, finally, a more general version of our algorithm that considers multiple fragment types. In section “Results and discussion”, we compare the performance of the proposed symmetric difference scoring model with the widely used shared peaks count scoring model. We consider experimental mass spectrometric data from synthesized peptides of known sequences (SWATH Gold Standard dataset [9]). The proof-of-concept implementation is available under a BSD license [10] and we plan to integrate it into the OpenMS framework [11].

## Problem definition

### Preliminary data cleaning

A peptide is composed of a chain of amino acids and, additionally, an oxygen and two hydrogen atoms. The mass of an uncharged peptide is the sum of its amino acid masses and the mass of the additional H$_2$O molecule (18 Dalton, [12]). In our exposition, we deal with the mass *M* that is the sum of the amino acid masses of the peptide, where the H$_2$O mass has already been subtracted. Moreover, let the set *X* represent the masses measured in the third step of the experiment including both 0 and the mass *M*.

### Notation

We represent a peptide as a string $\texttt {S}$ of characters (amino acids) of an alphabet $\Sigma$. Each character $\texttt {a}\in \Sigma$ has its own mass $m(\texttt {a}) \in \mathbb {R}^+$. For a string $\texttt {S} = \texttt {a}_1\ldots \texttt {a}_{\texttt n}$, we denote a substring by $\texttt {S}_\texttt {i,j}=\texttt {a}_\texttt {i}\ldots \texttt {a}_\texttt {j}$ for $1 \le i\le j \le n$. The mass of $\texttt {S}$ is the sum of its characters’ masses, i.e. $m (\texttt {S}) = \sum _{i=1}^{n} m{(\texttt {a}_{\texttt {i}})}$. The set $\text {Pre}(\texttt {S})$ of prefixes of $\texttt {S}$ contains every string $\texttt {S}_\texttt {1,i}$ for $1\le i \le n$ and the set $\text{Suf}(\texttt {S})$ of suffixes of $\texttt {S}$ every string $\texttt {S}_{\texttt j,n}$ with $1\le j\le n$. Both $\text{Pre}(\texttt {S})$ and $\text{Suf}(\texttt {S})$ additionally contain the empty string whose mass is zero. A *fragment* of $\texttt {S}$ is a prefix or a suffix of $\texttt {S}$. The *theoretical spectrum* of $\texttt {S}$ is the union of all fragment masses $\text{TS}(\texttt {S}) =$ $\{m(\texttt {T})\ |\ \texttt {T} \in (\text{Pre}(\texttt {S}) \cup \text{Suf}(\texttt {S})) \}$. A mass is *explained* by $\texttt {S}$ if it is in $\text{TS}(\texttt {S})$.

### Measuring the similarity of a string and a set of fragment masses

We want to find a string $\texttt {S}$ that explains a given set of fragment masses *X* as accurately as possible. We define the score of a string $\texttt {S}\in \Sigma ^*$ and a set of fragment masses *X* as an additive function

1 $$\begin{aligned} score (\texttt {S},X) = \sum _{m\in \text{TS}(\texttt {S})} f_*(m,X), \end{aligned}$$

where $f_*(m,X)\in \mathbb {R}$ indicates the score of a mass *m* that is explained by $\texttt {S}$ depending on whether *m* is in *X* or not. Past approaches [2, 3, 5] often considered the so-called shared peaks count, where one uses

2 $$\begin{aligned} f_{\text {scp}}(m,X) = |\{m\} \cap X| = {\left\{ \begin{array}{ll} 1&{\quad}\text { if } m\in X,\\ 0&{\quad}\text { if } m\notin X, \end{array}\right. } \end{aligned}$$

and variants of it. Conceptually, the shared peaks count computes the number of masses that are both in $\text{TS}(\texttt {S})$ and *X*.

In this paper, we do not want to only consider the masses explained by a string $\texttt {S}$ that are in *X*, but also the explained masses that are not in *X*. We aim to minimize the symmetric difference between TS($\texttt {S}$) and *X*. Equivalently, we can solve the problem of finding a string $\texttt {S}$ that maximizes $|\text{TS}(\texttt {S}) \cap X| - |\text{TS}(\texttt {S}) \setminus X|$. The reason is that for a fixed *X*, a chosen $\texttt {S}$ that maximizes the latter also minimizes the symmetric difference. Hence, we can define

3 $$\begin{aligned} f_{\Delta }(m,X) = |\{m\} \cap X| - |\{m\} \setminus X| = {\left\{ \begin{array}{ll} 1& {\quad} \text { if } m\in X,\\ -1&{\quad}\text { if } m\notin X. \end{array}\right. } \end{aligned}$$

### Problem definition

We can now formulate the de novo sequencing problem that we consider in this paper.

#### **The de novo sequencing problem.**

*Let*  $\Sigma$ *be an alphabet of characters, with a mass*  $m(\texttt {a})\in \mathbb {R}^+$ *for each* $\texttt {a}\in \Sigma$. *Given the peptide mass* $M\in \mathbb {R}^+$ *and a set* $X=\{x_i \in \mathbb {R}^+\ |\ i=1,\ldots ,k\}$ *of fragment masses, find a string* $\texttt {S}$ *of characters in* $\Sigma$ *with* $m(\texttt {S}) = M$ *that maximizes* $score(\texttt {S},X) = \sum _{m\in \text{TS}(\texttt {S})}f_{\Delta }(m,X)$.

## Algorithm

In this section, we present a dynamic programming algorithm for the de novo sequencing problem. Our algorithm builds on Chen’s algorithm [3, 13], a seminal graph-based algorithm for de novo sequencing that computes a string that maximizes the shared peaks count. We will briefly present Chen’s algorithm and then propose an algorithm that also accounts for masses that are explained by the computed string, but are not in the set of measured masses *X*.

Chen’s algorithm [3, 13] models the set *X* as a directed acyclic graph (*NC-spectrum graph*). A path in this graph represents a string. The problem of computing a string $\texttt {S}$ that maximizes $|\text{TS}(\texttt {S}) \cap X|$ is reduced to the *longest path avoiding forbidden pairs problem*, that is the problem of finding a longest path between two vertices *s* and *t*, such that at most one vertex of every given forbidden pair of vertices is used. This problem is NP-hard in general [14] and Chen’s algorithm [3, 13] solves the problem for a special structure of forbidden pairs on general directed acyclic graphs.

**Fig. 1:**
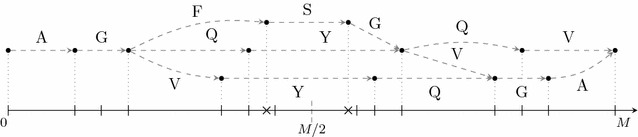
The set *X* is depicted on the real line (*bottom*): Masses in *X* are denoted by *vertical bars* and masses in $\bar{X}_M \setminus X$ by *crosses*. On the *top*, a subgraph of the NC-spectrum graph is shown. Every vertex represents a mass in $\bar{X}_M$ and an edge connects two vertices if their mass difference is equal to the mass of a string (only edges for strings of length 1 are shown)

The *NC-spectrum graph* (Fig. 1) is defined on the vertex set $\bar{X}_M = \big \{m, M{-}m\ |\ m\in X \big \}$. There is a directed edge from a vertex *v* to a vertex *w* if $w{-}v$ is equal to the mass of some string. A path from *v* to *w* represents one or multiple strings of mass $w{-}v$. For every vertex *a* traversed by the path, $a{-}v$ is a prefix mass of every string represented by the path. If a path from vertex 0 to vertex *M* traverses a vertex *a*, every string it represents explains both *a* (as a prefix mass) and $M{-}a$ (as the complementary suffix mass). It is sufficient to only traverse one of both complementary vertices *a* and $M{-}a$ to explain both.

To reduce the de novo sequencing problem to the longest path avoiding forbidden pairs problem, we assign weights to the vertices of the graph. Every vertex $v\notin \{0,M\}$ has weight $| \{v,M{-}v\} \cap X|$, namely the number of masses that are both in *X* and explained by traversing this vertex. The vertices 0 and *M* both have weight 1. The weight of a path is the sum of the weights of all vertices it traverses. If a path from 0 to *M* does not use both complementary vertices *a* and $M{-}a$ for some $a\in X \setminus \{0,M\}$, the weight of the path corresponds to the number of masses in *X* that are explained by a string represented by the path. On the other hand, consider a path that traverses both *a* and $M{-}a$ for some $a\in X \setminus \{0,M\}$. The weight of the path is higher than the number of masses in *X* that are explained, because masses in *X* that are explained by the vertex *a* and the vertex $M{-}a$ are counted twice. We exclude such paths by introducing forbidden pairs of vertices for all complementary vertices $\{a,M{-}a\}$ with $a\in X \setminus \{0,M\}$, such that a path uses at most one of both vertices. Note that each string of mass *M* is represented by a path from 0 to *M* avoiding forbidden pairs. The heaviest path avoiding forbidden pairs from 0 to *M* represents a string that maximizes $|\text{TS}(\texttt {S}) \cap X|$.

Chen et al. [3] propose a dynamic programming algorithm for computing the heaviest path avoiding forbidden pairs in this graph. For every pair of vertices $v\le M/2 < w$, the algorithm computes the maximal weight of any two paths from 0 to *v* and from *w* to *M*, such that no two vertices of a forbidden pair are both used by the paths. One path represents a prefix and the other one a suffix. In every step, the algorithm extends one of both paths until they can be concatenated, such that they represent a string of mass *M* that maximizes the size of $|\text{TS}(\texttt {S}) \cap X|$ among all strings $\texttt {S}$ of mass *M*.

**Fig. 2:**
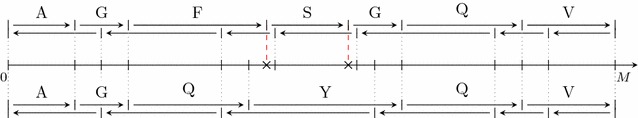
Two strings that maximize the number of explained masses in *X* (Fig. 1). While the upper string has two prefixes ($\texttt {AGF}$ and $\texttt {AGFS}$) explaining masses that are not in *X* (*crosses*), all masses explained by the lower string are in *X*

An example of the NC-spectrum graph is depicted in Fig. 1. For simplicity, we only consider edges connecting two vertices with a mass difference equal to the mass of a single character. In this example, two strings that maximize the number of explained masses in *X* are $\texttt {AGFSGQV}$ or $\texttt {AGQYQV}$ (Fig. 2). While the first string explains masses that are not in *X* (crosses), all explained masses of the second string are in *X*. We are only interested in the second string that minimizes the symmetric difference. At first sight, one might think that Chen’s algorithm can be easily modified to additionally consider how many explained masses are not in *X*. However, this is not obvious as the algorithm needs to check in every extension step, whether an explained mass that is not in *X* has already been explained in a different way in a previous step.

### An algorithm that minimizes the symmetric difference

We propose the algorithm **DeNovo** $\Delta$ that solves the de novo peptide sequencing problem as defined in the previous section. The algorithm considers a directed acyclic *multigraph* $G=(\bar{X}_M,E)$. For every pair of vertices *v* and *w* in $\bar{X}_M = \big \{m, M{-}m\ |\ m\in X \big \}$ and for every string with mass $w{-}v$ there is a directed edge from *v* to *w* in *E* that is labeled with this string. Note that all edges are directed from the smaller to the larger mass. *G* is a multigraph, because there can exist multiple strings with equal mass, i.e. multiple edges can connect the same pair of vertices. We denote the label of an edge (*v*, *w*) by $l(v,w)$ and the concatenation of the edge labels of a path *P* by $l(P)$. A path in *G* from *v* to *w* represents a string with mass $w{-}v$.

**Fig. 3:**
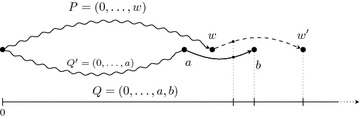
Two paths $P=(0,\ldots ,w)$ and $Q=(0,\ldots ,a,b)$. In every step, the algorithm extends the path ending in the smaller mass. In the next step, *P* is extended by an edge $(w,w')$. *Dotted lines* point towards masses on the real line (*bottom*) that are in $\text{TSe}(a,b),M)$, respectively $\text{TSe}((w,w'),M)$ (the complementary masses are omitted). As shown in this example, these two sets can have a non-empty intersection

The algorithm computes a string of mass *M* that minimizes the symmetric difference by iteratively extending two paths in *G*. Both paths start at vertex 0. One path represents a prefix and the other path a reversed suffix of the solution. The algorithm extends these paths until they end in two vertices *v*, respectively $M{-}v$. Then, the corresponding prefix and the reversed suffix can be concatenated to a string of mass *M*. In every step, the algorithm extends the path that represents the substring of smaller mass. In this way, the two corresponding substrings have similar masses throughout the execution of the algorithm. Let $P=(0,\ldots ,w)$ and $Q=(0,\ldots ,a,b)$ be two paths with $w\le b$ and $w+b \le M$ after some extension steps (Fig. 3). We know that $a\le w$, as the algorithm extends in every step the path ending in the smaller mass. If *a* would be larger than *w*, the algorithm would not have extended the subpath $Q'$ of *Q* ending in *a* by the edge (*a*, *b*) in a previous step, but *P* instead (by some other edge). Based on this observation, **DeNovo** $\Delta$ can update the number of explained masses that are in *X*, respectively not in *X*, efficiently while extending the paths.

We define the set of masses that are explained by the two paths *P* and *Q* as *partial theoretical spectrum*

$$\begin{aligned} \text{PTS}(P,Q,M) = \,& \{m{(\texttt {T}}), M{-}m({\texttt {T}})\,|\, {\texttt{T}}\in {\text{Pre}}({l(P)}) \cup {\text{Pre}}({l(Q)})\}. \end{aligned}$$

The partial theoretical spectrum of *P* and *Q* contains all masses that are explained by the prefix $l(P)$ and the reversed suffix $l(Q)$ for a given total mass *M*. Every mass in the partial theoretical spectrum of $P=(0,\ldots ,w)$ and $Q=(0,\ldots ,a,b)$ with $a\le w\le b$ is either smaller or equal *b* or larger or equal $M{-}b$.

Assume that the algorithm extends *P* by an edge $(w,w')$ in the next step (dashed edge in Fig. 3). By this extension, we explain the following set of additional masses

$$\begin{aligned} {\text{TSe}}((w,w'),M) = \{ m({\texttt{T}}) + w, \, M- (m({\texttt{T}}) + w)\ |\ {\texttt{T}} \in {\text{Pre}}(l{(w,w')}),\ m({\texttt{T}}) \ne 0\}.\end{aligned}$$

Note that we do not consider the empty prefix in $\text{Pre}(l(w,w') )$, because *w* and $M{-}w$ are already explained by *P*. Every mass in $\text{TSe}((w,w'),M)$ is larger than *w* and smaller than $M{-}w$. If the edge $(w,w')$ is labeled by a single character, $\text{TSe}((w,w'),M)$ contains only two masses, namely $w'$ and $M-w'$.

To compute the explained masses in *X* after the extension, we consider the masses that are explained by the edge $(w,w')$, but that have not been explained by *P* and *Q* (i.e. before the extension). The following invariant holds for every two paths $P=(0,\ldots ,w)$ and $Q=(0,\ldots ,a,b)$ computed by the algorithm: All masses, that are both explained by some outgoing edge $(w,w')$ of *w* and by *P* or *Q*, are in $\text{TSe}((a,b),M)$. That is, if a mass explained by $(w,w')$ is also explained by *P* or *Q*, then this mass is explained by the last edge (*a*, *b*) of *Q*. To see this, we first note that $\text{TSe}((w,w'),M)$ contains no mass that is explained by *P*, as every mass in $\text{TSe}((w,w'),M)$ is larger than *w* and smaller than $M{-}w$. Therefore, no mass explained by the edge $(w,w')$ has already been explained by *P*. Finally, by the extension rule of the algorithm it holds that $a \le w \le b$. Hence, every mass that is both explained by $(w,w^{\prime})$ and by *Q*, is explained by the last edge (*a*, *b*) of *Q*. Therefore, the invariant $\text{TSe}((w,w'),M) \cap \text {PTS}(P,Q,M) =$ $\text{TSe}((w,w'),M) \cap \text{TSe}((a,b),M)$ follows. This invariant holds for any two paths *P* and *Q* computed by the algorithm, even if *P* and *Q* share no explained masses, and for any outgoing edge $(w,w')$.

Thus, the algorithm does not have to remember all traversed vertices of the two paths in order to compute the newly explained masses after an extension. It is sufficient to remember the last two vertices of each of the paths, namely *w* and $w'$, respectively *a* and *b*. The set of newly explained masses of the last extension step is $\text{TSe}((w,w'),M) {\setminus}\text{TSe}((a,b),M)$. We define the additional score of this extension as

4 $$\begin{aligned} \text{gain}((w,w'),(a,b))= \sum _{m \in (\text {TSe}((w,w'),M) \setminus \text {TSe}((a,b),M))} f_{\Delta }(m,X). \end{aligned}$$

We compute a string with mass *M* that minimizes the symmetric difference with dynamic programming. We define a two-dimensional table *T* with |*V*| rows and |*E*| columns, where *V* denotes the set of vertices and *E* the multiset of edges of *G*. An entry *T*[*w*, (*a*, *b*)] contains the maximum score of any two paths $P=(0,\ldots ,w)$ and $Q=(0,\ldots ,a,b)$, i.e.

5 $$\begin{aligned} T[w,(a,b)] = \max _{P,Q} \Big \{ \sum _{m\in \text{PTS}(P,Q,M)} f_{\Delta }(m,X)\Big \}, \end{aligned}$$

where the maximum is taken over all paths $P=(0,\ldots ,w)$ and all paths $Q=(0,\ldots ,a,b)$ in *G*. We only consider an entry *T*[*w*, (*a*, *b*)] if $a\le w\le b$ and $w+b\le M$.

**Fig. 4:**
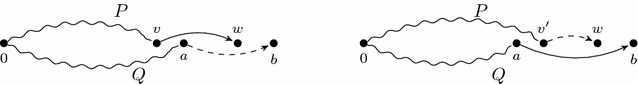
Computation of *T*[*w*, (*a*, *b*)]. Either a path $P=(0,\ldots ,w)$ ends with an edge (*v*, *w*) with $v \le a$ (*left*) or it ends in an edge $(v',w)$ with $v'>a$ (*right*)

By considering the invariant described above, we can compute the value of *T*[*w*, (*a*, *b*)] given the values of all entries *T*[*x*, (*c*, *d*)] with $x < w$ or $x=w$ and $c<a$ as follows (Fig. 4): Let $P=(0,\ldots ,w)$ and $Q=(0,\ldots ,a,b)$ with $a\le w \le b$ be the two paths that maximize the score among all paths ending in *w* and (*a*, *b*). We consider all incoming edges of *w* and distinguish two cases. Either the last edge of *P* starts at a source vertex that is at most as large as *a* or at a source vertex that is larger than *a*. In the former case, a subpath of *Q* was extended by the edge (*a*, *b*) in the last extension step before reaching *P* and *Q*. Hence, for an edge (*v*, *w*) with $v\le a$, we consider the value of *T*[*a*, (*v*, *w*)], that is the maximum score of any two paths ending in *a* respectively (*v*, *w*), and add the additional score of (*a*, *b*), i.e. $\text{gain}((a,b),(v,w))$. In the latter case, a subpath of *P* was extended by an edge ending in *w* in the last step before reaching *P* and *Q*. For an edge $(v',w)$ with $v'>a$, we add $\text{gain}((v',w),(a,b))$ to the value of $T[v',(a,b)]$. We consider all incoming edges of *w* in this way in order to cover all possibilities for reaching *P* and *Q*.

6 $$\begin{aligned} T[w,(a,b)]\, = \max {\left\{ \begin{array}{ll} \max \limits _{\begin{array}{c} (v,w) \in E,\\ v \le a \end{array}}\ \Big \{ T[a,(v,w)] + \text{gain}((a,b),(v,w))\Big \} \\ \max \limits _{\begin{array}{c} (v',w) \in E,\\ v' > a \end{array}}\Big \{ T[v',(a,b)] + \text{gain}((v',w),(a,b))\Big \}. \end{array}\right. } \end{aligned}$$

In the pseudocode of **DeNovo** $\Delta$ (Algorithm 1), we use a slightly different formulation from Eq. 6, as it simplifies the analysis of the algorithms’ time complexity. The algorithm first initializes every entry of table *T* by $-\infty$. To simplify the notation, we assume that *E* contains a loop edge (0, 0) and set $T[0,(0,0)] = 2$ (the empty string explains 0 and *M*). Then, the algorithm considers all vertices *v* in ascending order and for a vertex *v* all edges (*a*, *b*) with $T[v,(a,b)] \ne -\infty$ in ascending order of *a* and *b*. It extends the path ending in *v* by every outgoing edge of *v* and updates the corresponding entry in *T*. Once all entries have been computed, the optimal solution can be reconstructed starting from an entry $T[w,(v,M{-}w)]$ with maximal value among all vertices $v,w\in V$.

#### **Theorem 1**

*Given a peptide mass* $M\in \mathbb {R}^+$ *and a set* $X=\{x_i \in \mathbb {R}^+\ |\ i=1,\ldots ,k\}$ *of fragment masses, algorithm* **DeNovo** $\Delta$ *computes a solution for the de novo sequencing problem.*

#### *Proof*

We prove by induction that algorithm **DeNovo** $\Delta$ computes the entries of table *T* correctly. As base case, we see that the entries *T*[0, (0, *v*)] for all $(0,v)\in E$ are computed correctly. Assume that all entries $T[w',(a',b')]$ with $w'< w$ or $a' \le w'= w$ are correct. The next entry *T*[*w*, (*a*, *b*)] is either computed using an entry *T*[*a*, (*v*, *w*)] with $v\le a$ or an entry $T[v',(a,b)]$ with $a < v'$. Both entries are correct by the induction hypothesis. In the first case, $T[a,(v,w)] = \sum _{m\in \text{PTS}(P',Q,M)} f_{\Delta }(m,X)$ for some paths $P'=(0,\ldots ,a)$ and $Q=(0,\ldots ,v,w)$. A path *P* ending in *b* can be constructed by extending $P'$ with the edge (*a*, *b*). It remains to show that

$$\begin{aligned} T[w,(a,b)]&= \sum _{m\in \text{PTS}(P',Q,M)} f_{\Delta }(m,X) + \text{gain}((a,b),(v,w))\\&= \sum _{m\in \text{PTS}(P,Q,M)} f_{\Delta }(m,X). \end{aligned}$$

We denote the empty path by $P_0$. The set $\text{TSe}((a,b),M)\ \cap \ \text{PTS}(P',P_0,M)$ is empty, because every mass in $\text{PTS}(P',P_0,M)$ is in the interval [0, *a*] or $[M{-}a,M]$, but $a< m < M-a$ for every mass $m\in \text{TSe}((a,b),M)$. Moreover, $\text{TSe}((a,b),M)\ \cap \ \text{PTS}(P_0,Q,M) = \text{TSe}((a,b),M)\ \cap \ \text{TSe}((v,w),M)$ due to the fact that $v \le a \le w$. Therefore, no mass considered by $\text{gain}((a,b),(v,w))$ has already been considered when computing *T*[*a*, (*v*, *w*)]. We can prove the second case with a similar argument.

Let $\texttt {S}$ be an optimal string for the de novo sequencing problem. There are exactly two consecutive prefixes of $\texttt {S}$ with masses *v* and *w* such that $v \le M/2 < w$. The entry $T[M{-}w,(v,w)]$ is equal to $\sum _{m\in \text{PTS}(P,Q,M)} f_{\Delta }(m,X)$ for some paths $P=(0,\ldots ,M-w)$ and $Q=(0,\ldots ,w)$. Concatenating $l(P)$ and the reversed string of $l(Q)$ either results in $\texttt {S}$ or in another string $\texttt {S'}$ with $score(\texttt {S},X)=score(\texttt {S'},X)$, as $\texttt {S}$ is an optimal solution. $\square$

**Figure:**
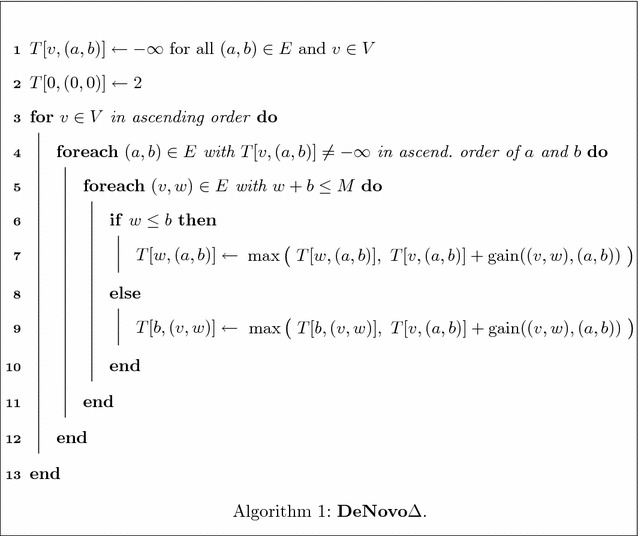


#### **Theorem 2**

*The time complexity of* **DeNovo** $\Delta$ *is in* $\mathcal {O}\left( |V|\cdot |E| \cdot d \cdot p\right)$ *, where d* *is the maximal out-degree of a vertex in* *G* *and* *p is the maximal length of an edge label.*

#### *Proof*

The table *T* can be initialized in $\mathcal {O}(|V|\cdot |E|)$ time. To compute an entry *T*[*v*, (*a*, *b*)], the algorithm considers all outgoing edges of *v*, that is at most *d* edges. The time for computing $\text{gain}(\cdot ,\cdot )$ depends linearly on the length of the label of an edge. Note that *G* is a multigraph and that there exists an edge from *v* to *w* for every permutation of the characters of *l*(*v*, *w*). As the maximal length of an edge label is *p*, which is bounded by $\mathcal {O}(M/\mu )$, where $\mu$ is the smallest mass of a character in $\Sigma$, the time complexity for considering an outgoing edge (lines 7 or 9) is in $\mathcal {O}(p)$. Thus, the runtime of **DeNovo** $\Delta$ is in $\mathcal {O}\left( |V|\cdot |E| \cdot d \cdot p\right)$. $\square$

When considering practical applications, the parameter *p* depends on the data quality rather than on the size of the input *X* and *M*. If we assume *p* to be a constant, there are only $\mathcal {O}(1)$ edges between two vertices and every vertex has only a constant out-degree. Hence, our algorithm matches the time complexity of Chen’s algorithm [3] unless the length of the edge labels grows asymptotically with the size of the input.

### Computing the *k* best solutions

In this section, we sketch how to find the *k* best solutions for the de novo peptide sequencing problem. Similar to the technique used in [15], we model the table *T* as a directed acyclic graph termed *matrix graph*. The edges in this graph correspond to all possible extension steps of our algorithm. The weight of an edge is equal to the additional score of the corresponding extension. A solution for the de novo sequencing problem corresponds to a path in this graph starting at the vertex representing the entry *T*[0, (0, 0)]. The score of the solution is equal to the weight of the path.

The matrix graph *MG* is a directed acyclic graph on vertices $V(MG) \subseteq (V\times E)$. For every entry *T*[*v*, (*a*, *b*)] with $a\le v \le b$ and $v+b \le M$, there is a vertex $v_{v,(a,b)}$ in *MG*. Every vertex $v_{v,(a,b)}$ has the following set of outgoing edges in *MG*:

$$\begin{aligned}&\left\{(v_{v,(a,b)},v_{w,(a,b)}) \ | \ (v,w)\in E,\ w \le b,\ w+b \le M \right \} \cup \\& \left \{(v_{v,(a,b)},v_{b,(v,w')}) \ | \ (v,w')\in E,\ w < b,\ w'+b \le M \right\}. \end{aligned}$$

Note that the edges defined above correspond up to renaming to the extension steps in lines 7 and 9 of **DeNovo** $\Delta$. The vertex $v_{v,(a,b)}$ represents paths ending in *v* and (*a*, *b*). The edges in the first set represent all extensions with edges (*v*, *w*) and $w\le b$, while the edges in the second set represent all edges $(v,w')$ with $w>b$. The weight of each of these edges is $\text{gain}((v,w),(a,b))$, respectively $\text{gain}((v,w'),(a,b))$, i.e. the additional score of adding the corresponding edge to the path in *G* ending in *v*.

A vertex $v_{v,(a,b)}$ in *MG* is a *terminal vertex* if $v = M{-}b$. A terminal vertex represents two paths that cannot be extended anymore, as they represent a prefix and a reversed suffix with a combined mass equal to *M*. A path from $v_{0,(0,0)}$ to a terminal vertex represents two substrings that can be concatenated to a string $\texttt {S}$ of mass *M*. The sum of the edge weights of this path is equal to $score(\texttt {S},X)$. Therefore, a solution for the de novo sequencing problem corresponds to a longest path from $v_{0,(0,0)}$ to some terminal vertex in *MG*.

Similarly, the *k*-th best solution for the de novo sequencing problem corresponds to the *k*-th longest path from $v_{0,(0,0)}$ to a terminal vertex in *MG*. We can apply Eppstein’s algorithm [16] to compute the *k* longest paths. Eppstein’s algorithm [16] computes the *k* shortest paths connecting a pair of vertices *s* and *t* in a directed acyclic graph with *n* vertices and *m* edges in $\mathcal {O}(n+m+k)$ time. The algorithm outputs an implicit representation of the paths and the sequence of edges of a path can be listed in time proportional to the length of the path. The matrix graph is a directed acyclic graph and in order to compute the longest instead of the shortest paths, we multiply all edge weights with $-1$. As the matrix graph can have multiple terminal vertices, but Eppstein’s algorithm only computes paths between two given vertices, we add a dummy vertex to the graph and connect all terminal vertices to this dummy vertex by directed edges with weight 0. Then, we compute the *k* longest paths between $v_{0,(0,0)}$ and the dummy vertex in *MG*.

We can build *MG* while executing **DeNovo** $\Delta$ in time $\mathcal {O}\left( |V|\cdot |E|\cdot d \cdot p\right)$, where *V* is the set of vertices and *E* the multiset of edges of *G*, *d* is the maximal out-degree of a vertex in *G* and *p* is the maximal length of an edge label in *G*. The matrix graph has $\mathcal {O}(|V| \cdot |E|)$ vertices and $\mathcal {O}(|V| \cdot |E| \cdot d)$ edges. Hence, we can find the *k* best solutions for the de novo peptide sequencing problem in $\mathcal {O}(|V| \cdot |E| \cdot d \cdot p + k)$ time.

### The general de novo sequencing problem

In the previous section, we studied the de novo sequencing problem in a simplified version. We assumed that a mass in *X* corresponds exactly to the mass of the amino acid sequence of the measured fragment. In real experiments, a mass in *X* can have a small offset from the mass of its string as a peptide can split at different chemical bonds between two amino acids and can loose small neutral molecules (e.g. water, ammonia). In this section, we study a more general version of the de novo sequencing problem that considers such mass offsets with bounded maximal pairwise difference. We present a modified version of **DeNovo** $\Delta$ for this problem.

First, we formulate the general de novo sequencing problem for a given set of possible mass offsets. We define the extended theoretical spectrum of a string $\texttt {S}$ as the set of all fragment masses with all possible mass offsets. As the possible offsets for prefixes and suffixes can differ, the extended theoretical spectrum of a string $\texttt {S}$ is not equal to the extended theoretical spectrum of the reversed string of $\texttt {S}$. Therefore, our modified algorithm **DeNovo** $\Delta _{g}$ for the general de novo sequencing problem needs to distinguish the prefix and the suffix string.

An important difference to the simplified problem is that mass offsets can alter the order of masses in *X* with respect to the masses of the corresponding strings. This complicates the computation of the newly explained masses of an extension step. While Chen’s algorithm [3] cannot deal with mass offsets that alter the order of the masses with respect to the masses of the corresponding strings, our algorithm can handle a broader range of mass offsets. The order of the masses in *X* with respect to the masses of the corresponding strings does not change if the maximal difference of any two offsets is smaller than the smallest mass $\mu$ of a character in $\Sigma$. We propose an algorithm that handles offsets with a maximal difference smaller than $2\cdot \mu$.

We model the extended theoretical spectrum as follows. Let $O_p$ and $O_s$ be the sets of all possible mass offsets $\delta \in \mathbb {R}$ for a prefix fragment, respectively a suffix fragment. A prefix of a string $\texttt {S}$ with mass *m* explains all masses in $\text {OM}(m,M) =\bigcup _{\delta \in O_p} (m+\delta )\ \cup \ \bigcup _{\delta ' \in O_s} (M{-}m+\delta '),$ where *M* is the mass of $\texttt {S}$. The *extended theoretical spectrum* of a string $\texttt {S}$ is the set of all prefix and suffix masses with all possible offsets $\text{TS}_{x}(\texttt {S}) = \bigcup \nolimits _{\texttt {T}\in \text{Pre}(\texttt {S})} OM(m(\texttt {T}), m(\texttt {S})).$ The maximal mass offset difference of two sets $(O_p,O_s)$ is $\gamma = \max _{\delta \in (O_p\cup \, O_s)}(\delta ) - \min _{\delta '\in (O_p\cup \, O_s)}(\delta ')$. Two sets $(O_p,O_s)$ of mass offsets are $\alpha$ *-basic* if $\gamma < \alpha \cdot \mu$.

#### **The general de novo sequencing problem**

*Let* $\Sigma$ *be an alphabet of characters, with a mass* $m(\texttt {a})\in \mathbb {R}^+$ *for each* $\texttt {a}\in \Sigma$. *Given a set* $X=\{x_i \in \mathbb {R}^+\ |\ i=1,\ldots ,k\}$ *of fragment masses, a peptide mass*  $M\in \mathbb {R}^+$, *and* 2-*basic sets* $(O_p,O_s)$ *of mass offsets, find a string* $\texttt {S}$ *of characters in* $\Sigma$ *with* $m(\texttt {S}) = M$ *that maximizes* $score(\texttt{S},X) = \sum _{m\in \text{TS}_{x}(\texttt {S})}{f_{\Delta }(m,X)}$.

We can solve the general de novo problem by considering a multigraph $G_{x}=(V_{x},E_{x})$. In contrast to the multigraph *G* defined above, $G_{x}$ contains up to $|O_p| + |O_s|$ vertices for each mass in *X*. For every $m\in X$, we consider every offset $\delta$ in $(O_p\cup O_s)$, assume that *m* is the mass of a fragment with offset $\delta$ and add a vertex with the corresponding prefix mass to the graph. The multiset of edges is defined in the same way as for the multigraph *G*. A path in $G_{x}$ from vertex 0 to vertex *M* corresponds to a string of mass *M*. In the same way as **DeNovo** $\Delta$, our algorithm for the general de novo sequencing problem **DeNovo** $\Delta _{g}$ extends two paths representing a prefix and a reversed suffix. In every step, the algorithm extends the path representing the string with smaller mass. The extension of a path by an edge $(w,w')$ explains the masses

$$\begin{aligned}&{\text{TSe}}_x((w,w^{\prime}),M) \,= \\ & \quad \left\{ \begin{array}{ll} \left\{ {\text{OM}}(w+m(\texttt{T}),M)\ |\ \texttt{T} \in {\text{Pre}}(l(w,w^{\prime})),\ m(\texttt{T}) \ne 0\ \right\}\ & {\text{if}} \ (w,w^{\prime})\ {\text{is added to}} \\ &{\text{the prefix path}}, \\ \left\{ {\text{OM}}(M-(w+m(\texttt{T})),M)\ |\ \texttt{T} \in {\text{Pre}}(l(w,w^{\prime})),\ m(\texttt{T}) \ne 0 \right\}\ &{\text{otherwise.}} \end{array}\right. \end{aligned}$$

Note that we distinguish prefixes and suffixes, as the extended theoretical spectrum is not necessarily symmetric. The set of newly explained masses by extending a path by an edge $(w,w')$ given the last edge (*a*, *b*) of the second path is

$$\begin{aligned}& {\text {New}}((w,w^{\prime}),(a,b))= \\ & \quad{\left\{ \begin{array}{ll} {\text {TSe}}_x((w,w^{\prime}),M) \setminus \left(\text {OM}(w,M) \cup {\text {OM}}(M-a,M) \cup {\text {TSe}}_x((a,b),M)\right) \ &{}{\text {if (w,w}}^{\prime}{\text{) is added to}} \\ &{}{\text {the prefix path}}, \\ {\text {TSe}}_x((w,w'),M) \setminus \left({\text {OM}}(M-w,M) \cup {\text {OM}}(a,M) \cup {\text {TSe}}_x((a,b),M)\right )\ &{}{\text {otherwise.}} \end{array}\right. } \end{aligned}$$

**Fig. 5:**
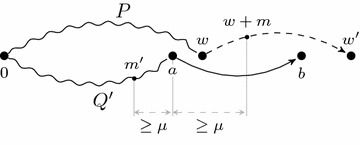
Extension of a path $P=(0,\ldots ,w)$ that represents a prefix by an edge $(w,w')$. The mass of the first character of the label of $(w,w')$ is *m*. Any mass $m'$ traversed by $Q=(0,\ldots ,a,b)$ before the source vertex *a* of the last edge (*a*, *b*) is at least $2\mu$ smaller than $w+m$

It is necessary to remove the masses in $\text {OM}(w,M)$, respectively $\text {OM}(M{-}w,M)$, even if the masses explained by the substring with mass *w* are not considered in $\text{TSe}_x((w,w'),M)$. This is due to the fact, that we consider 2-basic sets of mass offsets, where $\text {OM}(w,M) \cap \text {OM}(w',M)$ is not necessarily empty. Consider Fig. 5 for an illustration of the set of newly explained masses. The path *P*, which represents a prefix, is extended by an edge $(w,w')$. Let *m* be the mass of the first character of $l(w,w')$. The masses explained by $w+m$ in $\text{TSe}_x((w,w'),M)$ might also be explained by *w*. However, *m* cannot explain any masses that are explained by some mass $m'$ traversed by the other path before the source vertex *a* of the last edge (*a*, *b*), as the mass difference of $w+m$ and $m'$ is at least $2\mu$.

**DeNovo** $\Delta _{g}$ computes an optimal path in $G_{x}$ in the same fashion as **DeNovo** $\Delta$ described above. The algorithm can compute a solution for the general de novo sequencing problem in time $\mathcal {O}\left( |V_x|\cdot |E_x| \cdot d \cdot p \cdot |O| \right)$, where *d* is the maximal out-degree of a vertex in $G_{x}$, *p* is the maximal length of an edge label, and $|O|=|(O_p\cup O_s)|$ is the number of possible mass offsets.

### Scoring functions

A scoring function for the de novo sequencing problem compares the theoretical spectrum of a string $\texttt {S}$ with the experimental spectrum measured by the mass spectrometer. In the previous sections, we considered very intuitive scoring functions that count the number of masses in $\text{TS}(\texttt {S}) \cap X$ (shared peaks count, $f_{\text {scp}}$), respectively in $\text{TS}(\texttt {S}) \Delta X$ (symmetric difference, $f_\Delta$). These scoring functions do not consider any other information about the measured masses, such as the signal intensity, the type of the fragment, etc.

There exist several, more evolved scoring functions [4, 5] that consider, for example, the signal intensity $I(m)\in \mathbb {R}^+$ of each mass $m\in X$ measured by the mass spectrometer. Instead of only counting the number of explained masses that are measured in the experiment, the signal intensities of these masses are summed up. That is, a weighted shared peaks count with

7 $$\begin{aligned} f_{\text {wscp}}(m,X) = {\left\{ \begin{array}{ll} I(m) &{\quad}\text { if } m\in X,\\ 0 &{\quad}\text { if } m\notin X \end{array}\right. } \end{aligned}$$

is maximized. The intuition for this scoring function is that one prefers to explain fragment mass with high intensities, as the intensity corresponds to the abundance of the fragment and as low-intensity signals are more likely to originate from contaminants or measurement noise.

A weighted variant of the symmetric difference scoring function can be defined analogously. However, as we do not only consider measured masses, we define a constant penalty intensity $p\in \mathbb {R}$ for all masses $m\notin X$.

8 $$\begin{aligned} f_{w\Delta }(m,X) = {\left\{ \begin{array}{ll} I(m) &{\quad}\text { if } m\in X,\\ p &{\quad}\text { if } m\notin X. \end{array}\right. } \end{aligned}$$

In practice, one would rather use a non-constant penalty *p*(*m*) with some underlying model for predicting the signal intensity of a mass *m* based on the mass and the type of the fragment, the amino acids adjacent to the cleavage sites, and other factors.

To incorporate the weighted variant (Eq. 8) in the algorithm, it is sufficient to replace $f_\Delta ()$ by the weighted variant $f_{w\Delta }()$ in Eq. 4. No further modifications of the algorithm are necessary.

## Results and discussion

We implemented **DeNovo** $\Delta$ and studied the quality of its solution when using the shared peaks count scoring function and the symmetric difference scoring function. We chose **DeNovo** $\Delta$ rather than **DeNovo** $\Delta _{g}$ in our experiments to clearly expose the effect of the symmetric difference scoring function. While we are not primarily interested in runtime differences of both scoring functions, we observed that both algorithms have very similar performances (on average 5 s for one spectrum on an Intel Core i5-3317U CPU with 4 GB RAM, in some rare cases several minutes for one spectrum). We refer to Additional file 1: Figure S8 for a more detailed comparison of the running times. We note that the running times of state-of-the-art software packages as PepNovo [17], PEAKS [18], and especially Novor [19] are by magnitudes faster than the running times of our algorithm. However, we do not aim for an advanced software toolkit for de novo sequencing in this study, but rather propose a new fundamental scoring model that does not come at a substantial extra computational cost. The implementation is available under a BSD license [10].

We considered the DDA-mode experiments of 422 synthesized peptides that are part of the SWATH-MS Gold Standard (SGS) dataset (dataset PASS00289 at http://peptideatlas.org, [9]). First, we searched the spectra using the database search tool Comet [20] and a database containing only the sequences of the 422 synthetic peptides. The Comet search results were further validated using peptideprophet, which provides a statistical estimation for the false discovery rate [21]. We considered a peptide to be identified if the identification probability as returned by peptideprophet was higher than 90%. For our evaluation, we considered all spectra, where Comet was able to identify the expected synthetic peptide sequence. We did not consider spectra, where Comet reported a sequence with amino acid modifications or a sequence that was not ending with amino acid $\texttt {R}$ or $\texttt {K}$, as the current implementation of our algorithm is not able to consider such spectra. If Comet identified a peptide in multiple spectra, we considered all of them for our comparison, as it is not clear how to choose one of these spectra as the representative for the peptide. In total, we considered 944 spectra for our evaluation. We considered the raw profile data and implemented the merging algorithm proposed in [6] to reduce the size of the graph (i.e. centroiding). We consider that our algorithm identified a peptide if it reported the same correct sequence identified by Comet as the best-scoring sequence.

**Fig. 6:**
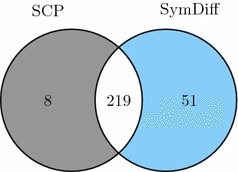
Number of peptides that where identified by **DeNovo** $\Delta$ when (i) maximizing the shared peaks count (SCP) and (ii) minimizing the symmetric difference (SymDiff)

Comet was able to identify 354 of the 422 synthesized peptides. Considering the shared peaks count (SPC), our algorithm identified 227 peptides, whereas it was able to identify 270 peptides considering the symmetric difference (SymDiff) scoring model (Fig. 6).

For a more detailed comparison, we first considered the position of the true sequence in the list of candidate solutions (sorted by their scores) and secondly the similarity of the best-scoring sequence with the true sequence. For the first comparison, our algorithm computed all solutions with a score of at least 90% of the maximum score. For the second comparison, we measured the similarity of two sequences by considering their sets of prefix masses. The *recall* of a reported sequence is the number of prefix masses it has in common with the true sequence divided by the number of prefix masses of the true sequence:

9 $$\begin{aligned} recall = \frac{\text {number of correct prefix masses}}{\text {number of prefix masses of the true sequence}}. \end{aligned}$$

**Fig. 7:**
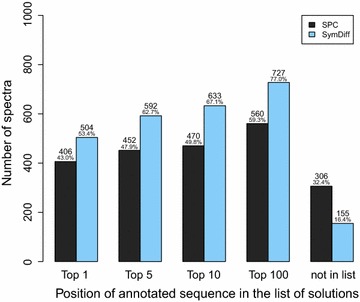
Position of the true sequence in the list of candidate solutions (sorted by score) when considering (i) the shared peaks count (SCP) and (ii) the symmetric difference (SymDiff) for the DDA measurements of the SGS dataset

Figure 7 shows the position of the true sequence (as annotated by Comet) in the list of candidate sequences (sorted by their score). The complete true sequence was among the top 10 sequences in 49.8% of the spectra considering the shared peaks count and in 67.1% of the spectra considering the symmetric difference.

**Fig. 8:**
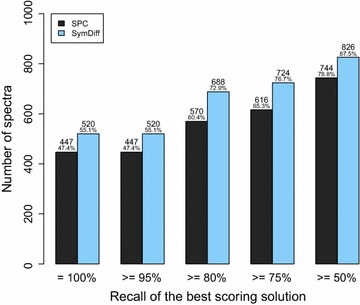
Recall of the best-scoring sequence reported by **DeNovo** $\Delta$ when considering the shared peaks count (SCP) and the symmetric difference (SymDiff) for the DDA measurements of the SGS dataset

Figure 8 depicts the similarity of the best-scoring sequence compared to the sequence identified by Comet. If there were multiple best-scoring sequences, we considered the one with the highest recall. In 60.4% of the considered spectra, our algorithm reported a sequence with a recall of at least 80% when considering the shared peaks count. Considering the symmetric difference, the best-scoring sequence had a recall of at least 80% in 72.9% of the considered spectra.

In a preliminary version of this paper [22], we considered the intensity-based variants of the shared peaks count and the symmetric difference scoring function defined in the previous section. Instead of considering the size of the sets $\text{TS}(\texttt {S})\cap X$ and $\text{TS}(\texttt {S})\setminus X$, we chose to sum up the intensities of the corresponding signals. The intensity-based variant of the shared peaks count is equivalent to the score proposed in [4]. However, the corresponding software PEAKS [18] uses additional features for scoring and is therefore not suitable for a comparison with our current implementation.

For this variant, one has to introduce a parameter *p*(*m*) for penalizing an explained mass *m* that is not in *X* when considering the symmetric difference. Setting $p(m)=0$ for all *m* is equivalent to considering the intensity-based weighted shared peaks count. We used $p(m)=-2500$ for all *m* in our experiments. We chose this parameter by empirically testing values ranging from $-10$ to $-5000$. The results for different values of *p*(*m*) appeared not to be very sensitive and other choices led to comparable results. For practical applications, it is more suitable to choose more evolved scoring functions, e.g. using a variable penalty parameter instead of a constant value for all masses.

We evaluated the DDA dataset using the intensity-based scoring function variants as well. While both the position of the annotated sequence and the recall of the best-scoring sequence improved using these scoring models (Additional file 1: Figures S1, S2), our algorithm was not able to identify more peptides with this variant. However, we were able to identify different peptides using these scoring models (Additional file 1: Figure S3).

Rather than penalizing equally all explained masses that are not measured, one can incorporate some model for predicting the signal intensities [23–25]. Similarly, in order to consider losses of neutral molecules or other types of fragments with mass offsets, one would need to define an appropriate penalty if the corresponding mass in the extended theoretical spectrum is not measured. For example, this penalty should depend on the type of the fragment and on whether a neutral loss is involved. Our algorithm can incorporate such aspects and gives us the possibility to develop more sophisticated scoring functions that model the fragmentation process more accurately.

Instead of using the raw profile data measured by the instrument and a simple merging algorithm [6], we additionally tested our algorithm on centroided (peak-picked) data. The data was peak-picked using the tool qtofpeakpicker [26]. Considering this preprocessed data, our algorithm was able to identify 237 peptides with the shared peaks count scoring function and 284 peptides with the symmetric difference scoring function. However, the identification rates declined considering the intensity-based variants of the scoring functions and we suppose that a more evolved model for penalizing explained masses that are not measured would be necessary to further improve the identification rates. We refer to the supplementary material for a more detailed comparison of the results for the raw profile and the preprocessed centroided data (Additional file 1: Figures S3–S7).

## Conclusion

In this paper we propose and study a new formulation of the de novo sequencing problem. Several previous approaches [3, 4, 6, 7] consider the set of masses that are both explained by a string and measured in the experiment. Although it has already been pointed out [6] that penalizing the fact that an explained mass is not measured improves the performance of algorithms for peptide identification, to the best of our knowledge the problem of minimizing the symmetric difference of the set of explained masses and the set of measured masses has not been studied before. We develop a dynamic programming algorithm that can compute both the best and the *k* best solutions for this new de novo sequencing problem. We conclude that without substantial extra computational effort, moving from shared peaks count to symmetric difference as optimization goal can improve the identification rates for de novo peptide sequencing.

## Additional file

**Additional file 1.** Supplementary Figures .
