# Supplementary material for: A better scoring model for de novo peptide sequencing: the symmetric difference between explained and measured masses
Source: Algorithms Mol Biol. 2017 May 11;12:12. doi: 10.1186/s13015-017-0104-1 (PMC5464308; doi:10.1186/s13015-017-0104-1)
Supplement: Supplementary file 1 — Additional file 1. Supplementary Figures [file 13015_2017_104_MOESM1_ESM.pdf]

A Better Scoring Model for De Novo Peptide Sequencing: The Symmetric Difference between Explained and Measured Masses – Supplementary Figures

Thomas Tschager[1*✉], Simon Rösch[1*], Ludovic Gillet[2] and Peter Widmayer[1]

[1]Department of Computer Science, ETH Zürich
[2]Department of Biology, ETH Zürich

A. Results for the intensity-based variants of the scoring models

In this section, we present some results for the evaluation of our algorithm with the intensity-based variants of the shared peaks count and the symmetric difference. We used a constant penalty intensity $p(m) = -2500$ for all m in all our experiments.

While both the position of the true sequence and the recall of the best-scoring sequence improved for both the shared peaks count and the symmetric difference, our algorithm was not able to identify more peptides. The algorithm identified 225 peptides for the intensity-based shared peaks count and 270 peptides for the intensity-based symmetric difference.

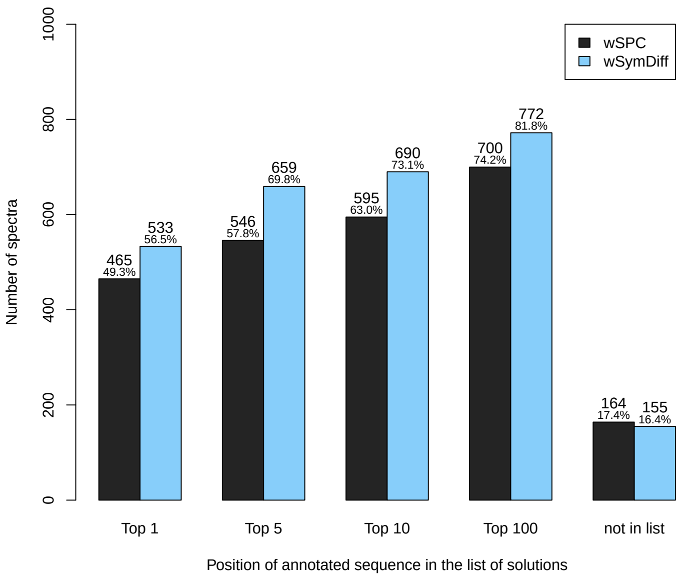

Figure 1: Position of the true sequence in the list of candidate solutions (sorted by score) when considering (i) the intensity-based variant of the shared peaks count (wSCP, cf. the definition of $f_{\mathrm{wscp}}(m, X)$ in the paper) and (ii) the intensity-based variant of the symmetric difference (wSymDiff, cf. the definition of $f_{w\Delta}(m, X)$ in the paper) with a penalty $p(m) = -2500$ for the DDA measurements of the SGS dataset.

✉ Corresponding author: tschager@inf.ethz.ch
* Equal contributor

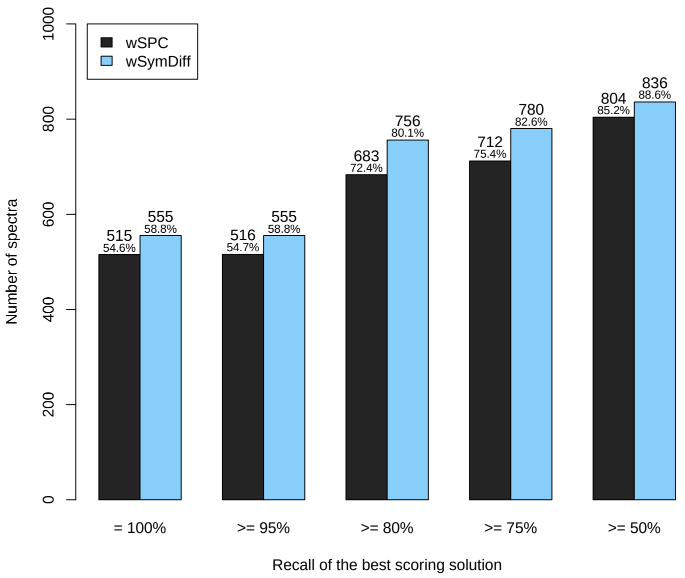

Figure 2: Recall of the best-scoring sequence reported by our algorithm when considering the intensity-based variant of the shared peaks count (wSCP, cf. the definition of $f_{\mathrm{wscp}}(m, X)$) and (ii) the intensity-based variant of the symmetric difference (wSymDiff, cf. the definition of $f_{w\Delta}(m, X)$ in the paper) with a penalty $p(m) = -2500$ for the DDA measurements of the SGS dataset.

B. Results for raw (profile) and preprocessed (centroided) spectra

We tested our algorithm on preprocessed, centroided mass spectra. Considering this preprocessed data, our algorithm was able to identify 237 peptides with the shared peaks count and 284 peptides with the symmetric difference. However, some peptides that the algorithm identifies in the raw data were not identified in the centroided data (and vice versa).

Note that the results do not improve when considering the intensity-based variants of the shared peaks count and the symmetric difference. We suppose that a fine-grained penalty function $p(m)$ would be beneficial to further improve the performance of the algorithm.

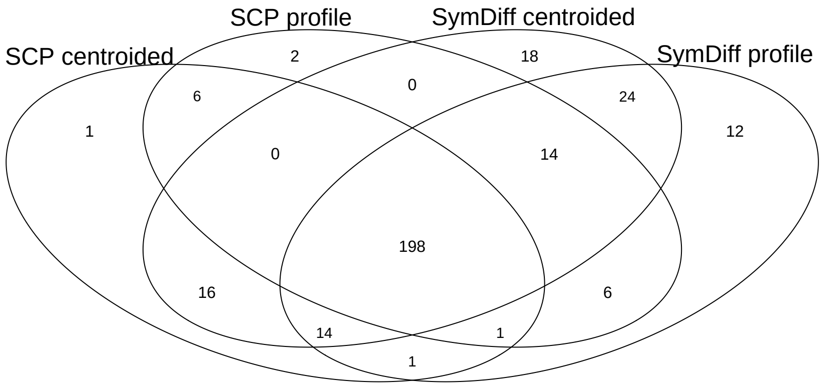

Figure 3: Number of peptides that where identified by our algorithm when (i) maximizing the shared peaks count (SCP) and (ii) minimizing the symmetric difference (SymDiff) using the raw profile (profile) spectra and the preprocessed spectra (centroided) of the DDA measurements of the SGS dataset.

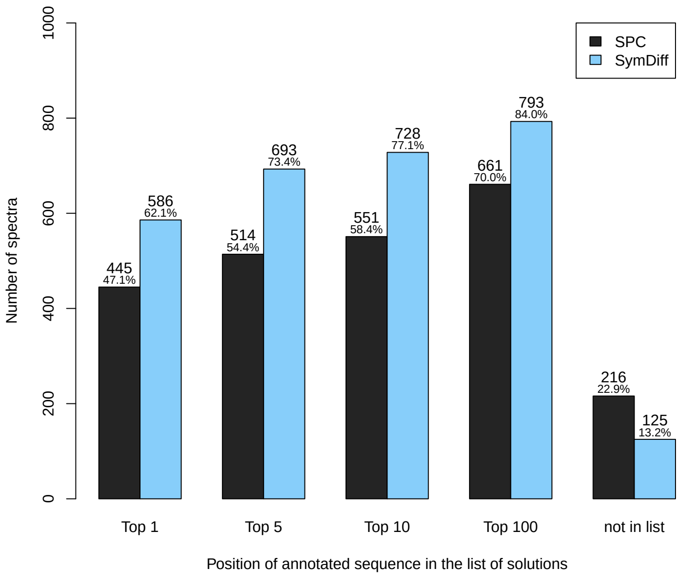

Figure 4: Position of the true sequence in the list of candidate solutions (sorted by score) when considering (i) the shared peaks count (SCP) and (ii) the symmetric difference (SymDiff) for the preprocessed (centroided, peak-picked) spectra of the DDA SGS dataset.

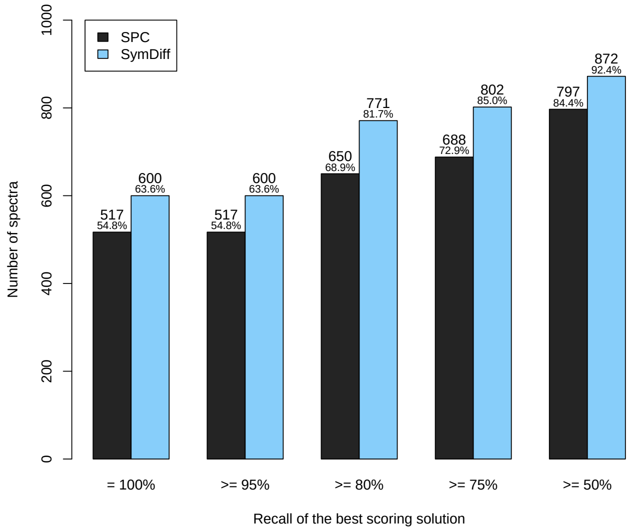

Figure 5: Recall of the best-scoring sequence when considering the shared peaks count (SCP) and the symmetric difference (SymDiff) for the preprocessed (centroided, peak-picked) spectra of the DDA SGS dataset.

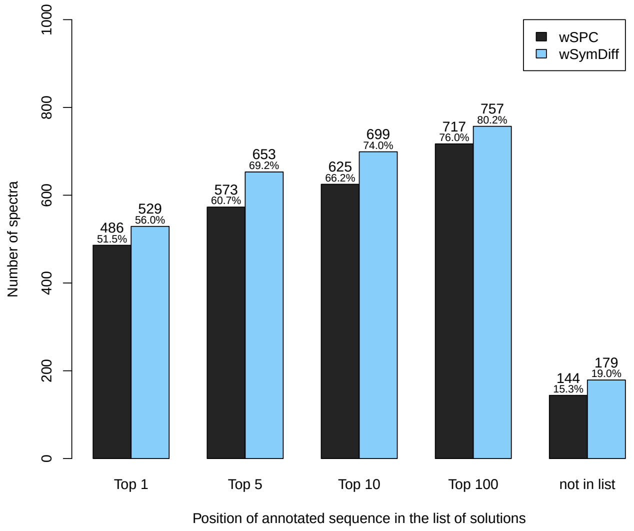

Figure 6: Position of the true sequence in the list of candidate solutions (sorted by score) when considering (i) the intensity-based variant of the shared peaks count (wSCP, cf. the definition of $f_{\mathrm{wscp}}(m, X)$ in the paper) and (ii) the intensity-based variant of the symmetric difference (wSymDiff, cf. the definition of $f_{w\Delta}(m, X)$ in the paper) with a penalty $p(m) = -2500$ for the preprocessed (centroided, peak-picked) spectra of the DDA SGS dataset.

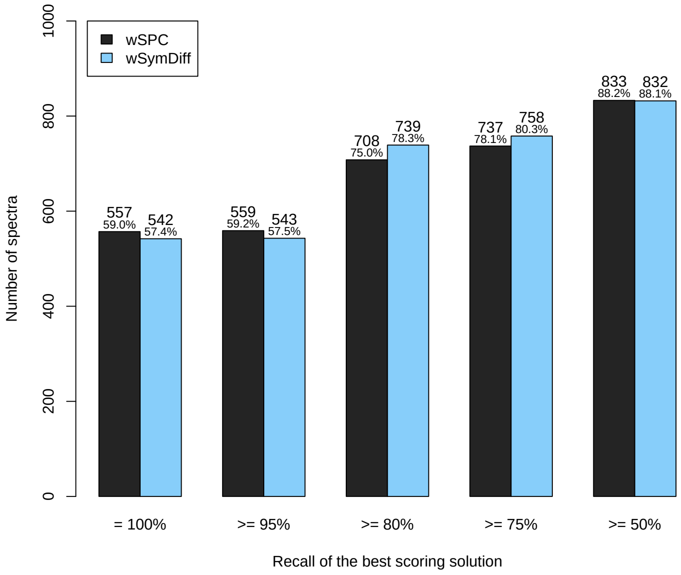

Figure 7: Recall of the best-scoring sequence reported by our algorithm when considering the intensity-based variant of the shared peaks count (wSCP, cf. the definition of $f_{\text{wscp}}(m, X)$) and (ii) the intensity-based variant of the symmetric difference (wSymDiff, cf. the definition of $f_{w\Delta}(m, X)$ in the paper) with a penalty $p(m) = -2500$ for the preprocessed (centroided, peak-picked) spectra of the DDA SGS dataset.

C. Running Time Analysis

In this study, we were not primarily interested in the runtime difference of the shared peak count model and the symmetric difference scoring model. However, we would like to note that we observed no significant difference for both scoring models. Our proof-of-concept implementation DeNovoΔ was able to analyze a single spectrum in 5 seconds on an Intel Core i5-3317U CPU with 4 GB RAM. Note that advanced de novo sequencing software toolkits are by magnitudes faster than our prototypical implementation. However, the runtime comparison (Supplementary Figure 8) indicates that considering the symmetric difference instead of the shared peak count does not come at a substantial extra computational cost.

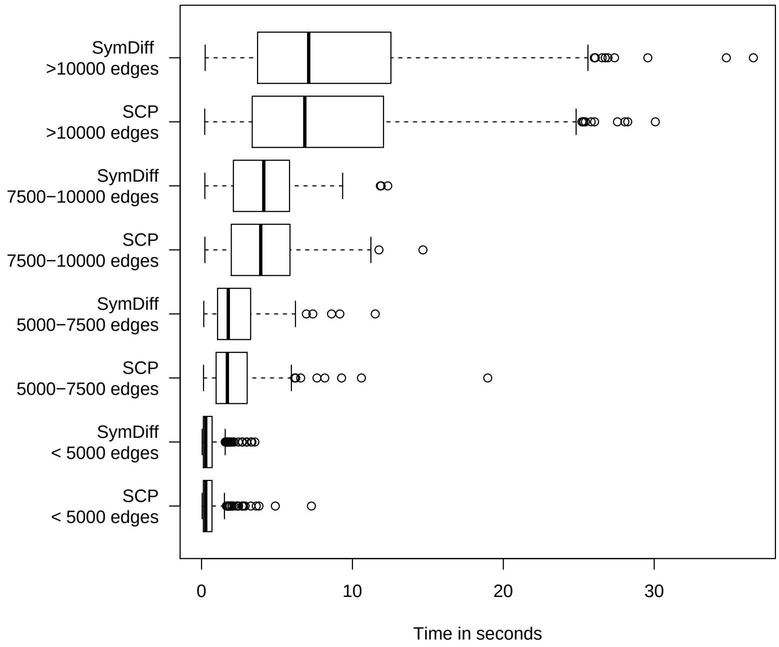

Figure 8: Comparison of the running times of DeNovoΔ for analyzing a single spectrum using the shared peak count (SCP), respectively the symmetric difference (SymDiff) scoring model. The distribution of the running times is shown for several intervals of graph sizes (number of edges). All running times were measured considering the preprocessed (centroided, peak-picked) spectra of the DDA SGS dataset.
